# Countrywide prevalence of critical drug interactions in Hungarian outpatients: a retrospective analysis of pharmacy dispensing data

**DOI:** 10.1186/s40360-019-0311-0

**Published:** 2019-05-31

**Authors:** Anna Somogyi-Végh, Zsófia Ludányi, Ábel Erdős, Lajos Botz

**Affiliations:** 1 Department of Pharmaceutics and Central Clinical Pharmacy, Clinical Centre, University of Pécs, Honvéd u. 3, Pécs, H-7624 Hungary; 2 IQVIA Solutions Services Kft., Váci út 1-3, Budapest, H-1062 Hungary

**Keywords:** Drug-drug interaction, Critical drug combinations, Prescription analysis, Patient safety, Pharmacy dispensing data

## Abstract

**Background:**

Drug-drug interactions (DDIs) present a significant source of adverse drug reactions. Despite being one of the commonly cited risks to patient safety, prevention of DDIs still poses a challenge to healthcare systems. The prevalence of DDIs can be used as a quality indicator for the safety of prescribing. With the analysis of drug utilization databases, real-world data on critical DDIs can be obtained. The aim of this study was to establish a list of critical DDIs and estimate their prevalence in the Hungarian outpatient population.

**Methods:**

Since there is no conclusive and generally accepted repository of high-risk DDIs, a systematic search of the literature for consensus-based lists was performed. Based on these results and their analysis with 5 interaction compendia, we propose a simple methodology to identify critical combinations. Present study focused on DDIs which are (1) of high clinical importance thus being most likely to cause significant harm if not detected, (2) well-supported by available evidence and (3) affect drugs which are routinely dispensed in the community pharmacy setting. A retrospective analysis of prescriptions filled between 2013 and 2016 was performed. The source of drug utilization data was the IQVIA’s national prescription fill database. The number of interacting drug pairs dispensed at the same time to the same patient was established.

**Results:**

After excluding drugs with low dispensing rates, the analysis covered 39 DDIs. The distribution of risk categories of the analysed DDIs was inconsistent among different drug interaction compendia. The total number of prescriptions filled varied between 173924449 and 176368468 per year. The prevalence of the selected potential DDIs ranged from 0.00 to 355.89 per 100000 prescriptions per year. There was significant variation between how the number of cases had changed for each DDI throughout the study period, no general tendency could have been described.

**Conclusions:**

There were 1.8 million cases of co-dispensing each year, where prescribers’ and community pharmacists’ role in recognizing and managing potentially serious interactions was or would have been critical. The method presented to identify high-risk DDIs can serve as a starting point for the much-needed improvement of routine interaction screening.

**Electronic supplementary material:**

The online version of this article (10.1186/s40360-019-0311-0) contains supplementary material, which is available to authorized users.

## Background

Drug-drug interactions (DDIs) present a significant source of adverse drug reactions (ADRs). A recent meta-analysis of 13 studies found that DDIs are responsible for approximately 1.1% of hospital admissions and 22.2% of all ADRs leading to admission are caused by DDIs [1]. Due to population aging and increasing polypharmacy, these ratios are expected to increase. According to a Scottish study, the proportion of adults dispensed ≥5 drugs doubled to 20.8% between 1995 and 2010, and the proportion of those dispensed ≥10 tripled to 5.8%. The prevalence of potentially serious DDIs went up to 13%, a more than twofold increase during the same period [2].

An enormous number of studies analysing potential interactions in different patient groups has been published in the past few decades. The prevalence of DDIs varies considerably depending on the study settings and applied methodology. This variability is well illustrated by the results of a recent review, where the rate of DDIs among elderly patients with multimorbidity ranged from 25 to 100% and the number of DDIs per 100 patients varied between 30 and 388 [3].

Despite being one of the commonly cited risks to patient safety, effective prevention of DDIs still poses a challenge to healthcare systems. Computerized interaction screening is widely implemented with the hope of reducing adverse drug events. However, issues related to inappropriate alerting, such as unclear clinical significance, database inconsistencies and alert fatigue are significant barriers to the meaningfulness of medication-related clinical decision support [4, 5]. Our previous work confirmed that interaction screening tools are fraught with contradictions and the information provided is moderately helpful in the clinical management of DDIs [6, 7]. Reasons for discrepancies among drug interaction compendia are summarized on Table 1.

**Table 1:** Reasons for discrepancies among drug interaction compendia [4, 8]

- Lack of standardized terminology to describe an interaction or its outcome	
- Heterogeneous criteria for severity classification	
- Inconsistent evaluation of evidence of the DDI	
- Variable reliance on sources such as non-English journal articles, postmarketing surveillance and product labeling	
- Inconsistent extrapolation of interactions to other drugs in the same class	
- Some knowledge bases intend to be highly inclusive, with an emphasis on breadth of coverage rather than clinical relevance due to medicolegal concerns	
- Various purposes intended for the database	
- Differences in the frequency of updates and latency of adopting new evidences	

Multiple attempts were made recently to address these challenges by a transparent and systematic assessment of underlying evidence and clinical relevance [9–13]. Based on these recommendations, a set of well-established, critical DDIs can be developed, the avoidance of which can be considered a minimum standard for healthcare providers.

The prevalence of DDIs can be used as a quality indicator for the safety of prescribing. For example, the number of patients with long-term prescriptions of any anticoagulant drug in combination with an oral NSAID is used as a quality indicator of prescribing in primary care in the OECD (Organisation for Economic Co-operation and Development) health statistics [14]. With the analysis of prescription drug claims and drug utilization databases, real-world data on critical DDIs can be obtained. Similar studies have been performed recently in Slovenia [15], Sweden [16] and Iran [17]. In these studies, 0.7–39 clinically relevant potential DDIs occurred per 1000 prescriptions [15, 17] and 3.8–9.3% of the population was exposed to such DDIs [15, 16]. Comparability of results is limited due to differences in methodology. In a previous study in Hungary, the rate of clinically significant interactions was found to be 2.7–3.6% based on the analysis of 1.2 million prescriptions [18]. No countrywide data on DDIs in the ambulatory population has been published as of yet. The aim of this study was to establish a list of critical potential DDIs and estimate their prevalence in the Hungarian outpatient population.

## Methods

### Selection of DDIs for analysis

**Fig. 1:**
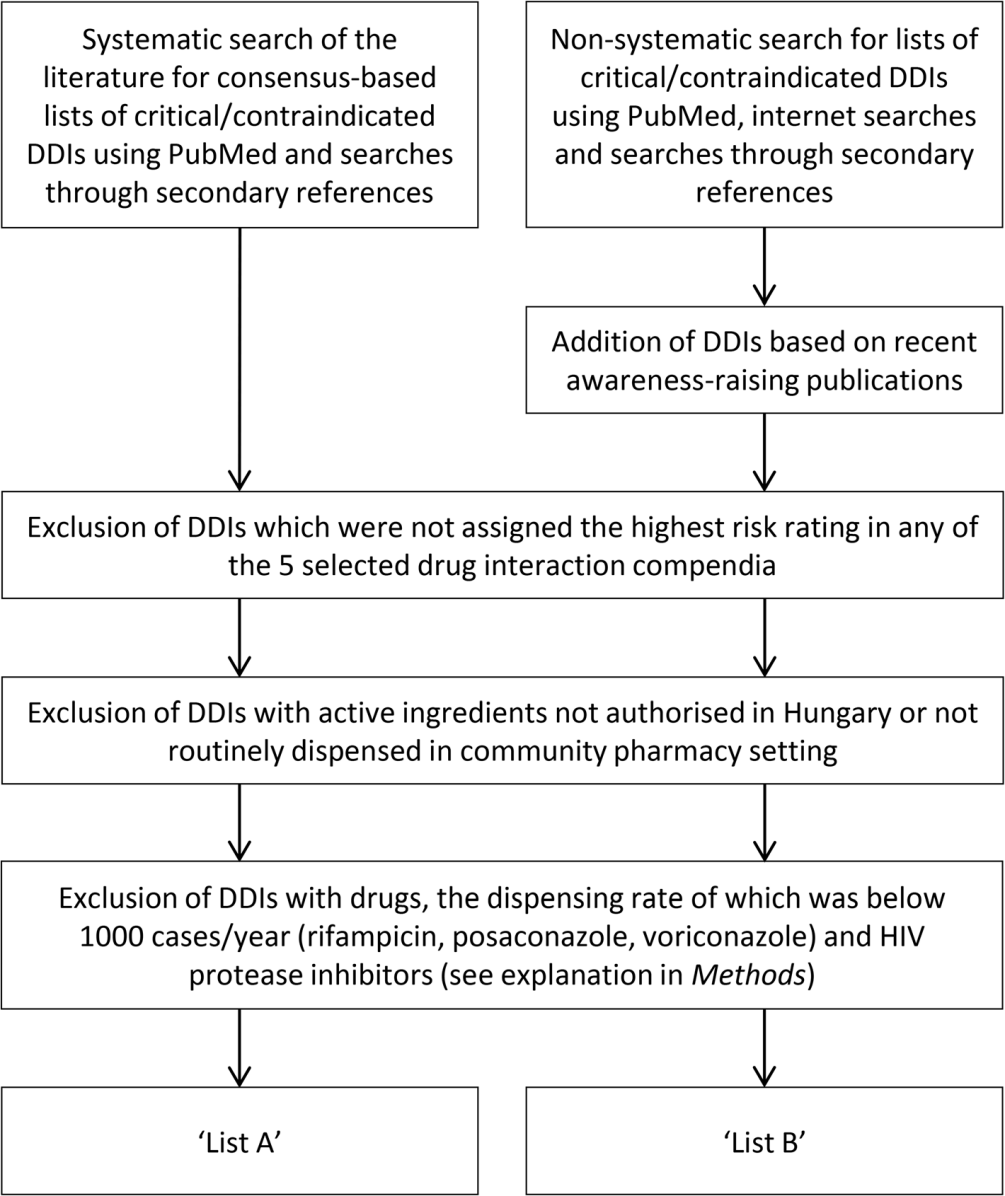
The process of selecting DDIs for analysis

There is no conclusive and generally accepted repository of contraindicated or high-risk DDIs, however, consensus recommendations for selecting DDIs for clinical decision support have been published recently [9, 19, 20]. We focused on DDIs which are (1) of high clinical importance thus being most likely to cause significant harm if not detected, (2) well-supported by available evidence and (3) affect drugs which are routinely dispensed in the community pharmacy setting. The process of selection of DDIs for the analysis is illustrated on Fig. 1.

‘List A’: A systematic search was conducted in the PubMed database using the search string ‘(drug interaction OR DDI) AND (panel OR consensus) AND (list OR contraindicated OR serious OR critical)’. After excluding irrelevant results and publications pertaining to special patient groups (e.g. paediatric, elderly or HIV patients), we identified 2 expert consensus lists of critical DDIs [21, 22]. To improve electronic systems and patient safety in the area of DDIs, the Partnership to Prevent DDIs was created in the US consisting of the Centers for Disease Control and Prevention (CDC), 3 academic institutions and a large pharmacy benefit manager company [21]. Using an expert panel and a standard evaluation tool, Malone et al. identified 25 clinically important DDIs between drugs dispensed in the ambulatory pharmacy setting. As a part of a larger project commissioned by the Office of the National Coordinator for Health Information Technology (ONC), a central repository of high-priority DDIs was created. In their report, a panel of experts identified 15 high-risk DDI subgroups for which warning should be generated in any medication-related decision support and electronic health record system [22]. After reviewing the references cited by or citing the 2 selected publications, we found one additional relevant article. Classen et al. [23] compiled a list of 7 DDIs that can be the starting point for the implementation of strong DDI checking, all of which had already been included in the ONC list, and was therefore disregarded.

‘List B’: To avoid omission of interactions of great practical importance, the high-risk classification of which is more debated, we decided to develop the more subjective ‘List B’. Intentional therapeutic co-administration of drug pairs on this secondary list is not uncommon in clinical practice since adverse consequences are generally less frequent. However, population-based studies indicate a significant increase in risks when used concomitantly, especially in patients with predisposing factors (see Discussion). Sources for the selection of DDIs on ‘List B’ included the list of top 10 particularly dangerous drug interactions in post-acute and long-term care published by AMDA - The Society for Post-Acute and Long-Term Care Medicine [24], the list of clinically important, common DDIs on the crediblemeds.org website [25] and the publication of Roughead et al. in which the authors established a list of DDIs considered serious by three or four references [26]. Three interacting pairs were added to the list based on a single recent publication because of the awareness raised in the medical community: the interaction between co-trimoxazole + angiotensin-converting-enzyme inhibitors [27], clarithromycin + colchicine [28, 29] and opioids and benzodiazepines [30]. In a case-control study of Fralick et al. in older patients receiving inhibitors of renin-angiotensin system, co-trimoxazole use was associated with an increased risk of sudden death [27]. In a 2010 analysis of colchicine-related deaths described in the literature, the US Food and Drug Administration identified 117 cases of fatal colchicine toxicity at therapeutic doses, 60 (51%) of which occurred in patients who were treated with clarithromycin at the same time [28]. In a retrospective study of 116 patients who were prescribed clarithromycin and colchicine, Hung et al. found that 9 (10.2%) of the 88 patients who received the two drugs concomitantly died [29]. With regard to the interaction between opioids and benzodiazepines, Sun et al. demonstrated a higher risk of opioid overdose associated with the increased concurrent use of benzodiazepines in a large sample of patients in the US [30].

In case of several group-based interactions we made some adjustments compared to the original lists to reflect the Hungarian pharmaceutical market. Moclobemide and rasagiline were not listed in the original publications, however, besides selegiline, these are the only available monoamine oxidase inhibitors (MAOIs) in Hungary, and therefore we decided to include them. Despite interactions affecting non-Vitamin K antagonist oral anticoagulants (NOACs) being also not included in the original lists, due to the steep increase in their use during the study period, we extended our analysis to 2 of their pharmacodynamic interactions. Contrary to the recommendations of Phansalkar et al. [22 we did not analyse all of the high risk QT prolonging agents simply because of the high number of possible combinations exceeding the scope of our study. Only considering drugs in ‘known risk of *Torsades de Pointes*’ category according to the CredibleMeds list [31], the number of possible combinations of drugs available in Hungary is as high as 210.

We decided to exclude drugs, the dispensing rate of which was below 1000 cases per year from our analysis since the statistical model used is expected to be less accurate in case of low prevalence. HIV protease inhibitors were excluded as well because of the relatively small number of HIV patients in Hungary and the special form of care they receive which includes atypical distribution of medicines, making the pharmacy panel data not representative for the ATC (Anatomical Therapeutic Chemical Classification) group J05AE.

### Analysis of risk ratings of the selected DDIs

The selected drug pairs were analysed in 5 drug interaction compendia: Lexicomp Drug Interactions (Lexi-Comp, Hudson OH, USA), Medscape Drug Interaction Checker (WebMD, New York, USA), Drugs.com (Drugsite Trust, Auckland, New Zealand), Janusmed Interactions (previously known as SFINX – Swedish Finnish Interaction X-referencing; Health and Medical Care Administration, Stockholm County Council, Stockholm, Sweden) and the Operational Classification of Drug Interactions (ORCA) based on the book ‘Top 100 Drug Interactions 2018 – A guide to patient management’ by PD Hansten and JR Horn [32]. If multiple alerts were displayed for the same drug pair, the one with the higher risk rating was the one taken into account. The grading systems of the selected DDI sources are summarised in Table 2.

**Table 2:** Classification of DDIs in the selected sources

Name	Categories
Lexicomp Drug Interactions	5 risk ratings:
	X – Avoid Combination,
	D – Consider Therapy Modification,
	C – Monitor Therapy,
	B – No Action Needed,
	A – No Known Interaction.
	3 severity ratings: Major, Moderate, Minor.
	4 reliability ratings: Excellent, Good, Fair, Poor.
Medscape Drug Interaction Checker	4 categories:
	Contraindicated,
	Serious – Use Alternative,
	Monitor Closely,
	Minor.
Drugs.com	4 categories:
	Major – Highly clinically significant. Avoid combinations; the risk of the interaction outweighs the benefit.
	Moderate – Moderately clinically significant. Usually avoid combinations; use it only under special circumstances.
	Minor – Minimally clinically significant. Minimize risk; assess risk and consider an alternative drug, take steps to circumvent the interaction risk and/or institute a monitoring plan.
	Unknown – No information available.
Janusmed Interactions	4 clinical relevance ratings:
	D – Clinically relevant interaction. The combination is best avoided.
	C – Clinically relevant interaction that can be handled e.g. by dose adjustments.
	B – Clinical outcome of the interaction is uncertain and/or may vary.
	A – Minor interaction of no clinical relevance.
	4-point scale for the level of documentation
ORCA	5 classes:
	Class 1 – Contraindicated (Risk of combination outweighs benefit),
	Class 2 – Provisionally contraindicated (Use only under special circumstances),
	Class 3 – Conditional (Assess risk and take actions if needed),
	Class 4 – Minimal Risk (Risk of adverse outcome appears small),
	Class 5 – No interaction (Evidence suggest that the drugs do not interact).

As an additional filter, DDIs which were not assigned the highest risk rating in any of the selected drug interaction compendia were excluded from further analysis.

### Study design

This study was a retrospective analysis of prescriptions filled between 1 January 2013 and 31 December 2016 in Hungarian community pharmacies.

### Data sources

The source of drug utilization data was the IQVIA’s (IQVIA Holdings, Inc., Durham, NC, USA, formerly known as QuintilesIMS) national prescription fill database. In this database, data is generated for a weekly period with a linear regression model method using three sources: the public database of the National Health Insurance Fund of Hungary (Hungarian acronym: NEAK), IQVIA’s wholesaler and retail pharmacy panel data. During the study period approximately 500–550 pharmacies were included in the pharmacy panel. The total number of Hungarian pharmacies within this timeframe varied between 2350 and 2390 according to the Hungarian Central Statistical Office [33]. A pharmacy profiling survey (location, opening hours, etc.) was used to refine the regression model.

IQVIA’s database is widely recognized as a representative source of countrywide drug utilization data, however, the details of the regression model are trade secret and we are not at liberty to disclose it. Several control mechanisms are in place to verify validity of data, including outlier detection and both random check and periodic comparison of transaction-based pharmacy panel data with IQVIA’s wholesaler (“sell-in”), ex-factory and NEAK data on product and sub-market (e.g. EphMRA Anatomical Class [34]) level.

### Data analysis

The number of cases was defined as the number of interacting drug pairs dispensed at the same time to the same patient. Co-dispensed drug pairs were identified by ATC code, transaction block identifier and anonymous patient identification code. All available brands of the affected active ingredients were taken into account, including both single-ingredient and combination forms. Products for topical use were excluded from the analysis.

Change in co-dispensing rates during the study period was analysed by using a simple linear regression model. Interacting pairs with low prevalence of co-dispensing (< 100 cases per any year) were not further analysed. 4-year change of data was only quantified for drug pairs with a good fit of the linear model (R^2^ ≥ 0.7). With this rather simple method our goal was not to precisely describe trends of change in prevalence – doing that would obviously require many more data points –, but to highlight interactions where an apparent increase or decrease transpires.

Interaction rates were calculated for each DDI as the number of prescriptions co-dispensed with an interacting drug divided by the total number of prescriptions for the object or precipitant drug. We report potential DDI rates per 1000 prescriptions involving the object or precipitant medication. For example, if Drug 1 and Drug 2 were co-dispensed in 5000 cases and the total number of Drug 1 prescriptions was 500000 in the same year, then the DDI rate would be 5000/500000*1000 = 10 per 1000 prescriptions for Drug 1.

## Results

### List of critical DDIs

After excluding interactions with drugs not marketed in Hungarian community pharmacies (e.g. irinotecan, pethidine), drugs with low dispensing rates (rifampicin, posaconazole, voriconazole) and HIV protease inhibitors, our analysis covered 39 DDIs. The final version of ‘List A’ included a total of 19 DDIs representing 140 ATC pairs, while ‘List B’ stood for 20 interactions described by 123 ATC pairs.

### Analysis of risk ratings of the selected DDIs

**Fig. 2:**
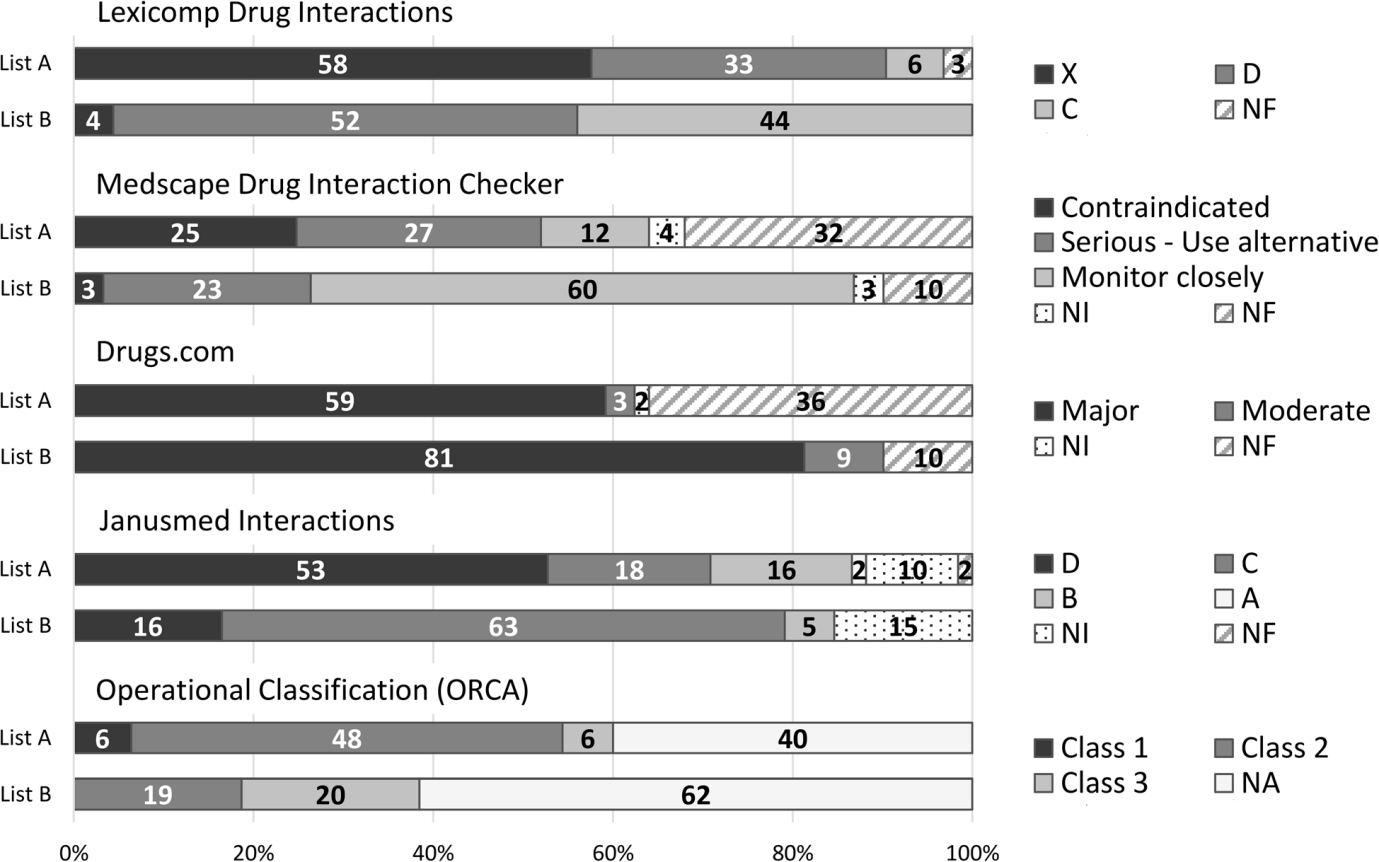
The distribution of risk categories of DDIs on ‘List A’ and ‘List B’ in %. NF – one or both of the drugs could not be found in the database, NI – no interaction identified, NA – no information available in the book [32]. See explanations for risk categories in Table 2

The distribution of risk categories of interactions on ‘List A’ and ‘List B’ are illustrated in Fig. 2 which is a good demonstration of how diverse an impression different databases give of the same set of interactions – due to differences in classification. However, the incompatibility of grading systems used in different compendia (see Table 2) limits the comparability of results. Drug interaction compendia of today usually focus more on the clinical manageability, rather than the severity of the potential outcome. From the 5 DDI sources used in our analysis, only Lexicomp has a separate rating system for both. Except of a few DDIs (Vitamin K antagonists + NSAIDs, theophylline + fluvoxamine), all interaction in Lexicomp with a risk rating of X or D had a severity rating ‘Major’ and all interactions with risk rating C were categorized as ‘Moderate’ severity. The ratio of interactions in the highest risk category was significantly higher among DDIs on ‘List A’ compared to ‘List B’ in all sources other than Drugs.com. However, if considering only the successful searches (i.e. when the input of both drugs was possible), the ratio of ‘Major’ DDIs compared to other risk categories was about 2% higher among interactions on ‘List A’ in this database too.

### Total number of prescriptions

The total number of prescriptions filled in 2013, 2014, 2015 and 2016 was 173924449, 174845548, 176115765 and 176368468, respectively.

The number of prescriptions for each interacting drug is available in an additional file [see Additional file 1].

### Cases of co-dispensing medications with critical DDIs

The number of cases, prevalence per 100000 prescriptions and change between 2013 and 2016 are shown in Tables 3 and 4. Drug 1 represents the object drug, while Drug 2 the precipitant drug (if applicable). The prevalence of the selected potential DDIs ranged from 0.00 to 61.68 per 100000 prescriptions per year on ‘List A’ and 0.00–355.89 on ‘List B’. The total prevalence of DDIs on ‘List A’ varied between 172 and 186 thousand cases per year, the co-dispensing of coumarin anticoagulants with NSAIDs and MAO-inhibitors with amphetamine derivatives being the most and least frequent DDIs, respectively. The zero prevalence of the latter is not surprising since amphetamine derivatives are typically prescribed for children and adolescents while MAOIs are mainly taken by older adults in Hungary, therefore being practically no overlap between the users of these two drug groups. The aggregate prevalence of DDIs on ‘List B’ was one order of magnitude higher with 1.60–1.66 million cases per year.

**Table 3:** Prevalence of the selected DDIs (‘List A’)

Drug 1 - Drug 2	Potential adverse consequence of the interaction	Prevalence per 100000 prescriptions (Number of cases)				R^2^	Change 2013–2016 (%)	Interaction rate*			
		2013	2014	2015	2016			2013	2014	2015	2016
Nitrates^a^ - PDE-5 inhibitors^b^	Hypotension	0.06 (95)	0.05 (82)	0.06 (97)	0.08 (135)	LP	LP	0.04, 1.03	0.04, 0.81	0.05, 0.71	0.07, 0.90
MAO inhibitors^c^ - SSRIs^d^	Serotonin toxicity	4.09 (7118)	4.21 (7365)	4.19 (7382)	3.96 (6980)	0.07	DNF	44.92, 3.74	46.96, 3.92	49.51, 3.93	46.03, 3.72
MAO inhibitors^c^ - Tricyclic antidepressants^e^	Serotonin toxicity	0.32 (551)	0.26 (446)	0.36 (631)	0.53 (933)	0.67	DNF	3.48, 3.51	2.84, 2.94	4.23, 4.13	6.15, 5.52
MAO inhibitors^c^ - Narcotic analgesics^f^	Serotonin toxicity	2.02 (3516)	2.16 (3770)	2.41 (4245)	2.41 (4252)	0.90	+ 21	22.19, 2.50	24.04, 2.52	28.47, 2.89	28.04, 2.90
MAO inhibitors^c^ - Triptans^g^	Serotonin toxicity	0.01 (22)	0.01 (13)	0.01 (13)	0.01 (16)	LP	LP	0.14, 0.38	0.08, 0.21	0.08, 0.18	0.10, 0.21
MAO inhibitors^c^ - Amphetamine derivatives^h^	Serotonin toxicity	0.00 (0)	0.00 (0)	0.00 (0)	0.00 (0)	LP	LP	0.00, 0.00	0.00, 0.00	0.00, 0.00	0.00, 0.00
QT prolonging agents^i^ - QT prolonging agents^i^	Potentially lethal ventricular arrhythmia	6.50 (11301)	6.52 (11401)	6.23 (10963)	5.63 (9925)	0.76	−12	NA	NA	NA	NA
Vitamin K antagonists^j^ - NSAIDs^k^	Bleeding	44.53 (77442)	49.46 (86478)	57.10 (100563)	54.42 (95977)	0.77	+ 24	41.38, 11.23	46.66, 12.28	55.36, 13.75	53.94, 12.95
Vitamin K antagonists^j^ - Fibric acid derivatives^l^	Bleeding	16.02 (27854)	15.67 (27395)	15.13 (26639)	14.56 (25677)	0.98	−8	14.88, 38.12	14.78, 38.88	14.66, 38.54	14.43, 37.29
Vitamin K antagonists^j^ - Barbiturates^m^	Reduced anticoagulant effect	0.47 (823)	0.62 (1079)	0.58 (1020)	0.78 (1376)	0.81	+ 67	0.44, 15.54	0.58, 21.81	0.56, 16.73	0.77, 17.72
Simvastatin - CYP3A4 inhibitors^n^	Statin toxicity (e.g. rhabdomyolysis)	13.81 (24013)	11.14 (19471)	8.96 (15774)	7.33 (12921)	0.99	−46	18.89, 20.39	18.87, 16.49	18.95, 13.57	18.07, 12.52
Theophylline - Ciprofloxacin	Theophylline toxicity (e.g. seizures, hypotension)	2.81(4884)	2.65 (4626)	2.62 (4620)	2.19 (3870)	0.81	−21	5.33, 8.86	5.14, 8.24	5.25, 8.47	4.64, 7.80
Theophylline - Fluvoxamine	Theophylline toxicity (e.g. seizures, hypotension)	0.15 (261)	0.12 (200)	0.06 (111)	0.24 (414)	0.14	DNF	0.25, 7.97	0.20, 6.88	0.11, 4.49	0.45, 19.10
Tizanidine - CYP1A2 inhibitors^o^	Tizanidine toxicity (e.g. hypotension, drowsiness)	0.73 (1262)	0.70 (1228)	0.67 (1172)	0.72 (1262)	0.03	DNF	8.55, 1.23	8.57, 1.21	8.53, 1.19	7.87, 1.36
Alprazolam - Azole antifungals^p^	Benzodiazepine toxicity	2.15 (3739)	2.48 (4335)	2.42 (4260)	2.33 (4110)	0.26	DNF	0.71, 13.35	0.79, 14.61	0.80, 13.97	0.78, 13.98
Ergot alkaloids^q^ - CYP3A4 inhibitors^r^	Ergot toxicity	0.14 (244)	0.15 (260)	0.13 (224)	0.11 (196)	0.70	−19	2.95, 0.34	3.27, 0.35	2.96, 0.30	2.67, 0.30
Thiopurines^s^ - Xanthine oxidase inhibitors^t^	Thiopurine toxicity (e.g. bone marrow suppression)	0.17 (297)	0.21 (365)	0.27 (472)	0.31 (546)	0.99	+ 84	5.46, 0.13	6.36, 0.15	7.97, 0.18	8.75, 0.20
Methotrexate - Trimethoprim	Methotrexate toxicity (e.g. bone marrow suppression)	0.00 (6)	0.00 (7)	0.01 (11)	0.01 (9)	LP	LP	0.19, 0.02	0.23, 0.03	0.32, 0.04	0.27, 0.03
Digoxin - Clarithromycin	Digoxin toxicity	0.07 (115)	0.05 (83)	0.03 (56)	0.04 (65)	LP	LP	0.60, 0.27	0.49, 0.19	0.36, 0.12	0.47, 0.19
	Total	99.28 (172673)	101.84 (178055)	105.79 (186309)	100.18 (176686)	0.21	DNF				

LP – not calculated because of low prevalence (< 100 cases/year), DNF – not calculated because the linear model does not fit (R^2^ < 0.7)

*number of interaction cases per 1000 prescriptions of Drug 1 and Drug 2, respectively

^a^glyceryl trinitrate, isosorbide mononitrate

^b^avanafil, sildenafil, tadalafil, vardenafil

^c^selegiline, rasagiline, moclobemide

^d^citalopram, escitalopram, fluoxetine, paroxetine, sertraline, venlafaxine (not SSRI but similar pharmacological properties)

^e^amitriptyline, clomipramine, imipramine, maprotiline, trimipramine

^f^fentanyl, tramadol

^g^eletriptan, sumatriptan, zolmitriptan

^h^atomoxetine, methylphenidate

^i^twelve combinations of the following: amiodarone, ciprofloxacin, citalopram, clarithromycin, domperidone, escitalopram, haloperidol

^j^acenocoumarol, warfarin

^k^aceclofenac, acemetacin, dexibuprofen, diclofenac, indometacin, lornoxicam, meloxicam, naproxen, niflumic acid, nimesulide

^l^bezafibrate, ciprofibrate, fenofibrate, gemfibrozil

^m^phenobarbital, primidone

^n^amiodarone, clarithromycin, fluconazole, itraconazole, verapamil, diltiazem

^o^ciprofloxacin, fluvoxamine, mexiletine, propafenone, amiodarone, ticlopidine

^p^fluconazole, itraconazole

^q^ergotamine

^r^clarithromycin, fluconazole, itraconazole

^s^azathioprine

^t^allopurinol, febuxostat

**Table 4:** Prevalence of the selected DDIs (‘List B’)

Drug 1 - Drug 2	Potential adverse consequence of the interaction	Prevalence per 100000 prescriptions (Number of cases)				R^2^	Change 2013–2016 (%)	Interaction rate*			
		2013	2014	2015	2016			2013	2014	2015	2016
Potassium chloride - ACE inhibitors^a^	Hyperkalemia	352.04 (612284)	355.89 (622256)	334.14 (588480)	338.53 (597064)	0.46	DNF	373.23, 38.45	390.52, 39.05	385.01, 36.60	396.43, 36.35
Potassium sparing diuretics^b^ - ACE inhibitors^c^	Hyperkalemia	241.56 (420132)	247.91 (433452)	240.62 (423769)	246.54 (434824)	0.38	DNF	319.27, 27.79	336.72, 28.45	335.44, 27.41	348.54, 27.38
Potassium chloride - Potassium sparing diuretics^b^	Hyperkalemia	85.93 (149452)	86.99 (152093)	81.10 (142823)	82.82 (146061)	0.39	DNF	91.10, 113.57	95.45, 118.15	93.44, 113.05	96.98, 117.08
Sulfamethoxazole/ trimethoprim - ACE inhibitors^c^	Hyperkalemia	11.40 (19822)	10.64 (18603)	10.28 (18099)	9.86 (17394)	0.97	−12	64.28, 1.24	70.11, 1.17	65.97, 1.13	67.05, 1.06
Vitamin K antagonists^d^ - Platelet aggregation inhibitors^e^	Bleeding	93.62 (162828)	97.67 (170779)	102.49 (180508)	98.27 (173316)	0.53	DNF	87.00, 22.48	92.15, 23.83	99.36, 25.02	97.40, 24.71
NOACs^f^ - Platelet aggregation inhibitors^e^	Bleeding	0.77 (1334)	2.84 (4961)	7.18 (12651)	12.90 (22748)	0.96	+ 1605	23.25, 0.18	36.63, 0.69	51.11, 1.75	57.70, 3.24
NOACs^f^ - NSAIDs^g^	Bleeding	0.48 (830)	2.06 (3607)	5.08 (8944)	8.77 (15472)	0.97	+ 1764	14.47, 0.12	26.64, 0.51	36.13, 1.22	39.24, 2.09
Vitamin K antagonists^d^ - Amiodarone	Bleeding	17.65 (30690)	17.96 (31398)	18.02 (31741)	17.47 (30813)	0.03	DNF	16.40, 276.48	16.94, 279.31	17.47, 281.21	17.32, 267.92
Vitamin K antagonists^d^ - Azole antifungals^h^	Bleeding	0.56 (971)	0.67 (1175)	0.57 (998)	0.66 (1164)	0.23	DNF	0.52, 4.00	0.63, 4.52	0.55, 3.70	0.65, 4.48
Vitamin K antagonists^d^ - Sulfamethoxazole/ trimethoprim	Bleeding	1.51 (2618)	1.37 (2387)	1.26 (2216)	1.21 (2127)	0.96	−19	1.40, 8.49	1.29, 9.00	1.22, 8.08	1.20, 8.20
Vitamin K antagonists^d^ - Metronidazole	Bleeding	0.42 (730)	0.39 (678)	0.44 (770)	0.45 (798)	0.54	DNF	0.39, 5.27	0.37, 4.70	0.42, 5.39	0.45, 5.77
Vitamin K antagonists^d^ - Ciprofloxacin	Bleeding	2.36 (4107)	2.37 (4152)	2.50 (4408)	2.09 (3681)	0.19	DNF	2.19, 7.45	2.24, 7.40	2.43, 8.09	2.07, 7.42
Narcotic analgesics^i^ - Benzodiazepines^j^	Respiratory depression and sedation	101.23 (176057)	109.39 (191260)	112.79 (198647)	114.47 (201896)	0.91	+ 15	125.09, 27.06	127.73, 28.70	135.34, 30.64	137.90, 31.57
Verapamil - Beta-blockers^k^	Cardiovascular adverse effects (e.g. bradycardia)	6.14 (10677)	5.77 (10093)	5.45 (9593)	4.93 (8697)	0.98	−19	36.00, 3.22	38.03, 3.29	39.67, 3.32	39.18, 3.21
Carbamazepine - Clarithromycin	Carbamazepine toxicity, reduced clarithromycin effect	0.16 (274)	0.15 (256)	0.11 (199)	0.11 (197)	0.89	−28	0.54, 0.64	0.52, 0.57	0.43, 0.44	0.43, 0.56
Colchicine - Clarithromycin	Colchicine toxicity (e.g. pancytopenia)	0.00 (5)	0.00 (4)	0.02 (28)	0.01 (11)	LP	LP	0.10, 0.01	0.09, 0.01	0.61, 0.06	0.24, 0.03
Digoxin - Amiodarone	Digoxin toxicity	0.81 (1406)	0.75 (1318)	0.67 (1179)	0.49 (864)	0.92	−39	7.39, 12.67	7.82, 11.72	7.59, 10.44	6.27, 7.52
Digoxin - Verapamil	Digoxin toxicity	1.26 (2190)	1.10 (1927)	0.83 (1456)	0.64 (1121)	0.99	−49	11.51, 7.38	11.44, 7.26	9.37, 6.02	8.12, 5.05
HMG CoA reductase inhibitors^l^ - Ciclosporin	Statin toxicity (e.g. rhabdomyolysis)	0.08 (136)	0.08 (134)	0.10 (175)	0.08 (145)	0.22	DNF	0.03, 15.84	0.03, 15.50	0.04, 17.73	0.04, 14.10
Tamoxifen - CYP2D6 inhibitors^m^	Reduced tamoxifen effect	0.04 (70)	0.02 (43)	0.03 (60)	0.01 (22)	LP	LP	5.63, 0.10	3.78, 0.06	4.71, 0.08	1.68, 0.03
	Total	918.02 (1596613)	944.04 (1650576)	923.84 (1626745)	940.37 (1658416)	0.56	DNF				

LP – not calculated because of low prevalence (< 100 cases/year), DNF – not calculated because the linear model does not fit (R^2^ < 0.7)

*number of interaction cases per 1000 prescriptions of Drug 1 and Drug 2, respectively

^a^benazepril, captopril, cilazapril, enalapril, fosinopril, lisinopril, perindopril, quinapril, ramipril, trandolapril

^b^amiloride, eplerenone, spironolactone

^c^captopril, enalapril, lisinopril, perindopril, ramipril

^d^acenocoumarol, warfarin

^e^acetylsalicylic acid, cilostazol, clopidogrel, prasugrel, ticlopidine

^f^dabigatran, apixaban, rivaroxaban

^g^aceclofenac, acemetacin, dexibuprofen, diclofenac, indometacin, lornoxicam, meloxicam, naproxen, niflumic acid, nimesulide

^h^fluconazole

^i^fentanyl, tramadol

^j^alprazolam, clonazepam

^k^metoprolol

^l^atorvastatin, simvastatin

^m^bupropion, duloxetine, fluoxetine, paroxetine

Sometimes changes in co-dispensing prevalence can easily be explained by the change in the overall frequency of prescribing one or both of the affected drugs. For instance, the number of cases where simvastatin and CYP3A4 inhibitors were co-dispensed had nearly halved in four years. The ratio of CYP3A4 inhibitor users, who also take simvastatin along with it had also dropped, while the rate of patients primarily using simvastatin and receiving a CYP3A4 inhibitor therapy has remained unchanged. These results can be explained by statins with lower potential for interaction becoming more commonly prescribed than simvastatin.

Other examples indicate a more complex change in prescribing patterns: In addition to the exponential increase in the co-dispensing of NOACs and platelet aggregation inhibitors, the ratio of NOAC users on concomitant antiplatelet therapy grew as well. The rate of interactions rose from 23 to 58 per 1000 NOAC prescriptions, despite the lack of a significant change in the total number of antiplatelet prescriptions during the same period.

Neither with List A, nor with List B is there a general tendency to be observed when looking at the total prevalence of interactions. However, the significant variation between how the number of cases had changed for each DDI throughout the study period makes the relevance of a sum total value questionable.

## Discussion

Prevention and management of drug-drug interactions is a challenging task for healthcare systems worldwide. Currently there is no established professional norm regarding which specific DDIs should come under scrutiny. Concentrating on drugs which are infamous for their promiscuous interacting potential (e.g. rifamycins, monoamine oxidase inhibitors, immunosuppressants) seems a reasonable approach; however, publications focusing on these are sometimes criticized for being disconnected from clinical reality [35]. As the “classic culprits” are being phased out from therapy, attention has shifted to drugs that have lower interacting potency, yet are much more frequently prescribed (e.g. HMG CoA reductase inhibitors, SSRIs, NSAIDs). In the present study both classes of DDIs were represented.

One of the key challenges when evaluating the clinical significance of DDIs is the lack of reliable data on the percentage of potential interactions resulting in an actual ADR. Through analysis of data from large clinical databases, valuable information about DDIs can surface which could not have been obtained using traditional approaches like clinical trials or spontaneous reporting. In this way the risk increased by co-administration can be quantified or specific predisposing factors can be identified. In a case series analysis of data from about 115,000 patients with upper gastrointestinal bleeding the authors determined the risk associated with different mono- and combination therapies. The risk of being diagnosed with upper gastrointestinal bleeding in combination therapy was greater than the simple sum of risks observed with monotherapies [36]. In another population-based study the risk of hyperkalaemia has been quantified with a combination of potassium-increasing drugs in presence and in absence of different risk factors (e.g. prolonged co-administration, renal function, diabetes) [37]. With novel approaches, such as coupling data mining and laboratory experiments even new DDIs can be discovered, as happened in the case of lansoprazole + ceftriaxone [38]. Timely integration of new knowledge into DDI compendia and summaries of product characteristics would be of paramount importance. Unfortunately, none of the three aforementioned publications are cited in any of the drug interaction compendia included in our analysis – except for Janusmed referencing Masclee et al.

The “Swiss cheese model” can be adapted to the problem of drug interactions in which the defences against adverse outcomes are represented by a series of slices [39]. Patient harm can only develop, if gaps in the defences line up. The risky combinations we analysed have already traversed the defences of the prescriber’s and the pharmacist’s knowledge as well as computerised screening. However, there are still several slices left which can prevent an actual ADR (e.g. patient factors, route of drug administration, monitoring).

Since data analysed in the study was collected from approximately one fourth of all community pharmacies in Hungary and since the linear regression model used multiple independent data sources to predict countrywide prevalence rates, the findings may well reflect the actual patterns of drug use in the country. As the self-reported use of prescribed medicines in Hungary is about the European average (49.8 vs. 48.6% in 2014) [40], this aspect does not limit the comparability of results with those of similar studies. However, since the clinical and economic burden of DDIs at the population level strongly depends on drugs available in the market and prescribing patterns in a local context, comparisons among different settings might be not relevant [41]. Despite of methodological differences, we think it is worth examine our results in contrast to similar Swedish data from 2010 [16]. Considering that the population of the two countries was similar at the time of the analyses (total population of Sweden in 2010: 9340682, Hungary in 2013: 9908798) [42] and that Holm et al. looked at a 4-month period, then dividing the number of co-dispensing cases in Hungary in 2013 by 3 allows for a crude comparison. Some DDIs seem to pose a bigger problem in Hungary, e.g. the one between potassium sparing diuretics + ACE inhibitors (40802 vs. 140044) or potassium-sparing diuretics + potassium salts (9902 vs. 49817), while the combination of organic nitrates with PDE-5 inhibitors was considerably more frequent in Sweden (1206 vs. 32). Examples for DDIs with approximately the same prevalence include warfarin + NSAIDs (4512 vs. 5595) and warfarin + low-dose acetylsalicylic acid (9382 vs. 8134).

The recent introduction of e-prescriptions in ambulatory care together with the increasing availability of unit-dose systems in hospital pharmacies in Hungary demands improvements to computerized interaction screening and clinical decision support systems; making them more effective at ensuring the safety of prescribing and dispensing. Over-alerting and consequent alert fatigue is still a key problem, a 2017 analysis revealed that 68% of DDI alerts are overridden by physicians, 62% of overrides being appropriate, meaning that 38% were not [43]. Implementation of national DDI rules may not adequately cover high-priority DDI pairs and may lack clinical efficiency in hospital settings [44]. In light of similar findings, some authors suggest to fundamentally question the premises of drug interaction alert systems [45]. More optimistic results have been published as well: the prevalence of potentially serious DDIs was significantly reduced after integration of the DDI database SFINX into electronic health records in primary care [46]. By now the selection and implementation of medication-related alerts into clinical decision support has become its own sub-field. Allowing healthcare professionals to tailor alerts to their needs together with the integration of individual patient characteristics in risk assessment are important components in reducing alert fatigue and improving computerized DDI detection in a meaningful way [35, 47].

## Limitations

Drug utilization data was only available for prescription-only medications, therefore co-dispensing with over-the-counter products was not evaluated. The limitation of interaction cases to co-dispensing at the same time instead of considering a broader time-frame might have resulted in the underestimation of risks. In a previous Hungarian study 50% of interacting drugs were dispensed at the same time [18]. Obtaining data regarding the number of affected patients was not possible, since the unit of the analysis was the prescription, not the individual subject. Also, we did not follow up the clinical outcomes for persons exposed to these potential interactions. We focused on a narrow set of DDs which are generally considered to be clinically significant, however, we did not assess de presence of factors increasing or mitigating the risk of an adverse outcome (e.g. dose, age, laboratory monitoring).

## Conclusions

Since DDIs are considered a predictable and preventable cause of ADRs, minimising the risks associated with exposure to potentially harmful drug combination is essential. The goal of preventive interaction screening is to ensure that no patient receives a potentially dangerous combination without previous evaluation and ongoing control of risks. Our motivation to perform this study was to obtain real-world data laying the groundwork for the much-needed development of routine interaction screening and DDI-related clinical decision support. The main question we wanted to answer was whether a preselected group of critical DDIs poses a significant danger to the Hungarian ambulatory population. Our results indicate that the problem of DDIs cannot be declared resolved. There are 1.8 million cases of co-dispensing each year, when prescribers’ and community pharmacists’ role in recognizing and managing potentially serious interactions is critical. Today, we have the technology to make interaction screening more effective. Reaching a consensus on an elementary set of interactions would be a great leap forward in improving patient safety, the method presented to identify critical DDIs can serve as a starting point for that.

## Additional file

Additional file 1: Number of prescriptions filled for the analysed interacting drugs. The total number of dispensings for each analysed drug/drug group. (XLSX 13 kb)

## Abbreviations

ADR: Adverse drug reaction
ATC: Anatomical Therapeutic Chemical Classification
DDI: Drug-drug interaction
MAOI: Monoamine oxidase inhibitor
NEAK: National Health Insurance Fund of Hungary
NOAC: Non-Vitamin K antagonist oral anticoagulant
NSAID: Nonsteroidal anti-inflammatory drug
ORCA: Operational Classification of Drug Interactions
